# Leveraging an advanced simulated moving bed approach to achieve 3-component separation for enhanced impurity removal in a non-affinity cation exchange capture step

**DOI:** 10.1371/journal.pone.0280760

**Published:** 2023-01-25

**Authors:** Serene W. Chen, Zi Ying Zheng, Farouq Bin Mahfut, Yuansheng Yang, Masahiro Ogino, Kazuo Okada, Kohei Sato, Wei Zhang

**Affiliations:** 1 Downstream Processing Group, Bioprocessing Technology Institute, Agency for Science, Technology and Research, Singapore, Singapore; 2 Cell Line Development Group, Bioprocessing Technology Institute, Agency for Science, Technology and Research, Singapore, Singapore; 3 Functional Materials Development, R&D Center, R&D and Engineering, Organo Corporation, Koto City, Japan; Michigan Technological University, UNITED STATES

## Abstract

One of the key challenges in downstream bioprocessing is to obtain products of high purity in a productive fashion through the effective removal of process and product related impurities. While a classical simulated moving bed (SMB) system operation can typically achieve a 2-component separation between the weakly bound impurities and target species, here we present an advanced SMB approach that can achieve a 3-component separation, including the removal of the strongly bound impurities from the target species. As a proof-of-concept, we demonstrate the enhanced removal of strongly bound host cell proteins (HCP) from the target monoclonal antibody (mAb) through the utilisation of the advanced SMB approach in a non-affinity cation exchange (CEX) capture step. In this way, 1 less polishing step was required to achieve the therapeutic requirements of < 100 ppm HCP and the overall process recovery was increased by ~ 6% compared to the corresponding process that utilised a batch CEX operation. The non-affinity CEX capture platform technology established through the utilisation of the advanced SMB approach presented here can potentially be further applied to address the downstream processing challenges presented by other challenging biotherapeutic modalities to yield a final target product with improved purity and recovery.

## 1. Introduction

One of the leading classes of biopharmaceutical therapeutics in today’s market is that of monoclonal antibodies (mAbs), with the mAb market size valued at USD 144 billion in 2020 and is expected to almost double by 2027 [1]. The establishment of robust manufacturing platform technologies is therefore important to ensure a smooth and rapid transition from the discovery of product candidates to clinical trials and commercial production. In recent years, remarkable developments in upstream processing have led to a steady and significant increase in mAb titers [2–5]. However, the high product titers, along with the concomitant increase in host cell proteins (HCP) and other impurities [6,7] often challenge the efficiency of traditional purification methods, resulting in a shift in the manufacturing costs from upstream processing towards downstream processing [2–4]. As such, it is critical for further developments in downstream processing to occur so as to ensure that products of high purity can be obtained in a productive manner.

**Fig 1:**
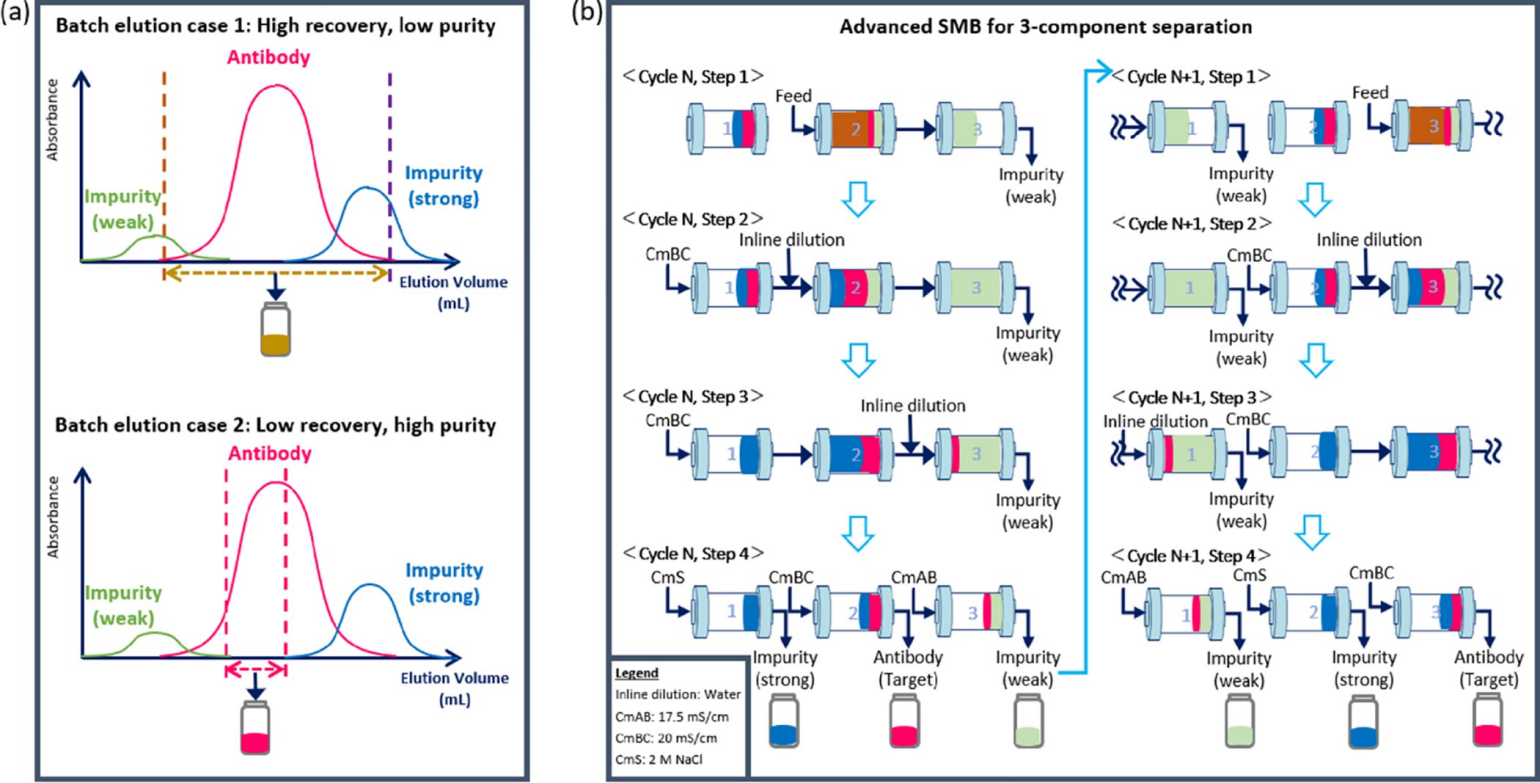
(a) High purity is often sacrificed at the expense of recovery and *vice versa* depending on whether the impure fractions containing target products are kept or discarded in a batch elution process. (b) While a classical SMB operation can typically achieve a 2-component separation between the weakly bound species and the target, here we present an advanced SMB approach that can achieve a 3-component separation (right) [18]. The feed, antibody target, strongly bound impurities and weakly bound impurities are illustrated in brown, pink, blue and green respectively. As recycling of impure fractions from the previous step is an important feature of the advanced SMB approach presented here, the relative position of each component is illustrated for each step in 2 representative cycles of the steady state phase. CmAB, CmBC and CmS correspond to different conductivities required to elute the weakly bound impurities, target and strongly bound impurities respectively as outlined in Table 1 and inline dilution was performed with MilliQ water.

One of the promising developments in this aspect is multi-column counter current chromatography. In single column batch chromatography, there is typically an under utilization of the resin, as the loading phase is stopped before the product starts to breakthrough, so as to prevent product loss. An increase in resin utilization can be achieved in multi-column chromatography systems by having a 2^nd^ column to capture the target compound that breaks through from the 1^st^ column. Another limitation in batch chromatography is that there is often a trade-off between purity and recovery, where impure fractions containing products may be collected or discarded accordingly in order to achieve high recovery or high purity (Fig 1A). This can however be at least partially circumvented by collecting high-purity fractions and recycling the portions where peaks overlap for another cycle of separation. An example of such a system is the simulated moving bed (SMB) system, where a two-component separation between the weakly bound species (raffinate) and the target component (extract) can typically be achieved in a classical operation [8]. Ternary SMB chromatography refers to a SMB system that can provide ternary separation, namely the separation of strongly bound species, weakly bound species, and target product in one chromatography. Some studies on ternary SMB chromatography designs and applications have been reported, although most of them either require laborious and costly cascade schemes such as two four-zone SMB units [9] or have mainly been applied to chemical separations [10].

**Table 1:** Optimised SMB CEX operation parameters applied on three 1.33 mL columns.

Step	Time (s)	Flow rate (mL/min)	Buffer conditions
1–1	1450	1.0	Feed: pH 6.0, 5 mS/cm CmAB: pH 6.0, 17.5 mS/cm CmBC: pH 6.0, 20.0 mS/cm CmS; pH 6.0, 2 M NaCl
1–2	180	2.0	
1–3	60	2.0	
1–4	250	2.0	

Another way of further lowering the cost of the downstream process is to utilize an alternative to Protein A resin as the capture step. Protein A chromatography is considered a ‘gold standard’ in mAb capture step purification due to its ability to yield mAbs with excellent purity and recovery, although this often occurs at the expense of high resin costs and longer processing times associated with a typical bind and elute resin chromatography step. The recent introduction of Fibro® technology for Protein A chromatography presents itself as an attractive alternative to Protein A resins by drastically increasing the overall operational productivity through providing optimal flow and binding dynamics [11]. The use of non-affinity resins as an alternative capture step has also been proposed as a resin cost reducing measure [12–14]. While products of high purity are typically more easily attainable through affinity capture steps due to the higher ligand-target specificity, strategies such as introducing additional salt wash steps have been proposed to enhance impurity removal in non-affinity methods [14].

While SMB technology has been applied to the Protein A capture step of mAb [15,16], its application to cation exchange (CEX) has been comparatively limited, where applications have been demonstrated for the purification of a model mixture of IgG/lysozyme [17] and as a capture step where achieving higher equipment utilization and productivity was the main aim [18]. Here, using an industrially representative Humira biosimilar mAb cell culture supernatant (CCS), we seek to develop an advanced SMB non-affinity CEX capture platform approach to achieve a 3-component separation, including that of the strongly bound impurities from the target species, so as to improve the final product purity and recovery. As a proof-of-concept, we demonstrate the enhanced removal of strongly bound HCP from the target mAb, leading to a reduction of 1 polishing step required to meet therapeutic requirements of < 100 ppm HCP and an improvement of ~6% in overall process recovery compared to the corresponding process utilizing the batch CEX operation.

## 2. Materials and methods

Unless otherwise stated, all buffers, salts and reagents were purchased from Merck Millipore.

### 2.1 Humira biosimilar cell culture production

Humira biosimilar were produced using a CHO K1 cell line, which was developed in-house in 14-day fed-batch cultures in 2 L bioreactors. The CHO K1 cell line was thawed and passaged three times in 30 mL of EX-CELL Advanced CHO Fed-batch medium supplemented with 6 mM of glutamine and 250 nM of MTX in 50 mL tubespins. Each fed-batch culture was started by inoculation of cells into 1500 mL of EX-CELL Advanced CHO Fed-batch medium supplemented with 6 mM of glutamine but without MTX. The inoculated viable cell density was 3×10^5^ cells/ mL. The cultures were maintained at 37°C, 50% dissolved oxygen, pH 6.9, and stirring at 150 rpm. 6% Cell boost 7a and 0.6% Cell boost 7b were fed into culture at day 3, 5, 7, 9 and 11. Samples were taken at day 3, 5, 7, 9, 11 and 14 for cell count using the Vi-Cell XR viability analyzer and quantification of antibody titer using Nephelometer. Major metabolites were analyzed at specified days by using Nova Bioprofile 100plus analyzer. When the concentration of glucose dropped to below 2 g/L, a specified volume of 45% glucose stock was added into fed-batch cultures in order to achieve final glucose concentration at 6 g/L. CCS was harvested at day 14 for purification. In each fed-batch culture, the maximal cell densities reached around 2.5 ×10^7^ cells/mL at day 9. The viabilities at the end of cultures were around 90%. The titers at the end of cultures were around 5 g/L as determined by the IMMAGE 800 immunochemistry system.

### 2.2 96-well plate screening for the determination of optimal conditions for column studies

The screening of optimal experimental conditions was performed in 96-well plates using central composite face-centered (CCF) design in MODDE 12.1 Design of Experiment (DoE) software. In order to eliminate differences in starting materials caused by precipitation at low pH and conductivity, CCS was titrated to pH 4.5 and incubated for 1 h at room temperature with stirring, followed by 0.22 μm microfiltration, before adjusting to the respective pH and conductivities for POROS XS (Thermo Fisher Scientific) CEX resin loading condition screening at a 5 mg/mL-R load. The 96-well plate screening of optimal conditions for polishing steps using POROS HQ (Thermo Fisher Scientific) anion exchange (AEX) resin, POROS Benzyl Ultra (Thermo Fisher Scientific) hydrophobic (HIC) resin and Capto MMC ImpRes (Cytiva) multimodal resin were performed sequentially at a 25 mg/mL-R load. The representative trends obtained from the contour plots generated were used to determine optimal column conditions and the materials obtained from the optimised column studies were used for the subsequent plate screening.

For all 96-well plate screening studies, 10 or 20 μL of resin was added to each well and equilibrated with equilibration buffer (300 μL x 3). CCS or the respective eluates at the required pH and conductivities were then added to each well and incubated under shaking conditions (1 h, room temperature, 500 rpm for all resins, except for Benzyl Ultra where 15 min incubation time was recommended by the product brochure [19]). When screening for optimal loading conditions in the bind and elute mode for POROS XS and Capto MMC ImpRes, 50 mM MES 1M NaCl at pH 6.5 and pH 6.0 were used as elution buffers respectively (200 uL x 2; 10 min, room temperature, 500 rpm). The removal of solution from each well was performed by centrifugation (1000 g, 1 min).

### 2.3 AKTA^TM^ chromatography

1 mL or 4 mL of resin were packed, as required, in Tricorn^TM^ series columns (Cytiva) with a diameter of 0.5 cm and 1.0 cm respectively. All columns had a bed height of 5.1 cm and all experiments were conducted on an ÄKTA^TM^ Avant 25 (Cytiva). The chromatography media used include POROS XS (Thermo Fisher Scientific), POROS HQ (Thermo Fisher Scientific), POROS Benzyl Ultra (Thermo Fisher Scientific) and Capto MMC ImpRes (Cytiva).

For the comparison of the effect of different CEX loading conductivities on the breakthrough curves, CCS was titrated to pH 4.5 and incubated for 1 h at RT with stirring followed by 0.22 μm microfiltration, before adjusting to pH 6 and the respective conductivities, so as to eliminate potential differences caused by precipitation at the low pH and conductivity. After ascertaining the optimal loading pH and conductivity as 6 and 5 mS/cm respectively, the pH and conductivity of all CCS was adjusted with acetic acid and water respectively, followed by passing through a SUPRAcap HP PDK5 depth filter (PALL, SC050PDK5) at 7.0 mL/min. The optimal processing volume was determined to be more than 2 L in order to obtain high recovery. The processing flow rate and volume were scaled linearly when a larger PDK5 depth filter (PALL, NP5LPDK51) was used. After sample loading, 50 mM MES pH 6, 5 mS/cm buffer was passed through the depth filter until the UV absorbance at 280 nm returned close to baseline and reached a plateau. For dynamic binding capacity (DBC) analysis, the breakthrough percentage was obtained by monitoring the percentage of monomer concentration in the flow through (FT) relative to that in the CCS.

### 2.4 SMB

Three 1.33 mL POROS XS (Thermo Fisher Scientific) columns were packed, with a diameter of 1.0 cm and a bed height of 1.7 cm each. The column length was unified for comparison with batch purification using CEX as a capture. All experiments were initially conducted on an Octave 10 chromatography system (Tosoh Bioscience Wisconsin Inc.) as previously reported [20]. To enhance the removal of HCPs that more strongly bound to CEX than antibodies, new operation method was created as shown in Fig 1B, which can be expected to separate the target and the strongly bound impurities. Table 1 shows the operating conditions. The flow rates and step times were determined based on the calculated trajectories of components, namely the weakly, moderately and strongly adsorptive components, so that each component ended at its corresponding extraction point. There was a slight difference between the calculated value and the measured value as band broadening was not taken into consideration in calculation, therefore, fine adjustments were made during subsequent process validation and optimization.

### 2.5 HPLC-Size Exclusion Chromatography (SEC) for antibody concentration and purity analysis

100 μL of 0.22 μm filtered samples were injected onto a TSK_gel_ G3000SW_XL_ column (7.8mm i.d. x 30cm; Tosoh Bioscience). The mobile phase buffer consists of 0.2 M L-arginine, 0.05 M MES, 5 mM EDTA, 0.05% sodium azide (w/w), pH 6.5. The flow rate used was 0.6 mL/min, with UV absorbance monitored at 280 nm. The amount of high molecular weight (HMW) and low molecular weight (LMW) species were calculated using the area of peaks which eluted at earlier and later retention times compared to the monomeric peak respectively, where HMW 1 and 2 refers to the 1^st^ and 2^nd^ peak observed before the monomeric peak and LMW 1 and 2 refers to the 1^st^ and 2^nd^ peak observed after the monomeric peak. The target monomeric concentration was determined based on the area under the peak as compared to a calibration curve obtained using standard samples.

### 2.6 Residual host cell protein analysis

The CHO HCP content was determined by the microtiter plate enzyme-linked immunosorbent assay (ELISA) 3^rd^ Generation CHO HCP kit (Cygnus Technologies), according to manufacturer’s instructions. Data acquisition was performed with the Gen5^TM^ software on a Synergy^TM^ 2 plate reader (BioTek).

### 2.7 Residual host cell DNA analysis

The CHO DNA content was measured using a QX200^TM^ Droplet Digital^TM^ PCR System (Bio-Rad Laboratories), according to manufacturer’s instructions. Briefly, samples were digested with proteinase K (0.2 mg/mL in 0.5% SDS, 16 h, 50°C), followed by inactivation (10 min, 95°C). DNA extraction from the resultant solution was subsequently performed using QIAamp^®^ viral RNA mini kit (Qiagen). The PCR reaction mixture was prepared by mixing the following together: ddPCR^TM^ supermix for residual DNA quantification (Bio-Rad Laboratories), ddPCR^TM^ CHO residual DNA quantification assay (Bio-Rad Laboratories), Xeno^TM^ VIC^TM^ primer probe mix (Applied Biosystems), Xeno^TM^ DNA control (Applied Biosystems) and the extracted DNA sample. The generation of the droplets was performed using the automated droplet generator (Bio-Rad Laboratories) and the 96-well PCR plate was heat-sealed with PX1^TM^ PCR plate sealer (Bio-Rad Laboratories). The PCR reaction (10 min at 95°C; 40 cycles of 30 s at 94°C followed by 1 min at 60°C; 10 min at 98°C) was performed with C1000 Touch^TM^ thermal cycler (Bio-Rad Laboratories). Data recording and analysis was performed with QuantaSoft analysis software (Bio-Rad Laboratories). The conversion of DNA copy number to DNA concentration was based on CHO host cell DNA standards (Applied Biosystems).

## 3. Results and discussion

### 3.1 Development of SMB CEX capture step based on batch CEX protocol

A 3-column advanced SMB approach is presented here (Fig 1B), with the capability of separating weakly, moderately and strongly adsorptive components [20]. One cycle comprises of 4 steps, where step 1 consists of passing the feed through two columns connected in series. The feed overloads the first column, and its breakthrough continuously loads onto the second column before switching to the second step. The elution buffer (CmBC) is then passed through three columns connected in series during the next two steps, with MilliQ water introduced through different inlet ports to achieve a conductivity corresponding to that of the wash buffer (CmAB) via inline dilution. In step 2, CmBC pushes out the components remaining in previous steps and separates the target substance and weakly adsorbed components using a salt concentration changed by dilution (CmAB). CmAB is the salt concentration set to separate the target substance and the weakly adsorbed component, and the weakly adsorbed component moves in the bed at a faster speed than the target substance and separates. After that, in step 3, the target substance and strongly adsorbed components are separated by CmBC, which is set for separation of the target substance and strongly adsorbed components. In step 3, the target substance moves in the bed at a faster speed than the strongly adsorbed component and separates. At this time, since the target substance and weakly adsorbed components have been separated in step 2, it is possible to recover the target substance with high purity in step 3. Fractions in which some weakly adsorbed components and target compounds and target compounds and strongly adsorbed components are mixed are left in the bed and separated again in the next cycle. Therefore, it is possible to improve the recovery rate while maintaining high purity. In step 4, the columns were washed with buffers of different conductivities respectively, and the weakly, moderately and strongly adsorptive components were eluted and collected in separate ports and impure fractions recycled in the next cycle.

**Fig 2:**
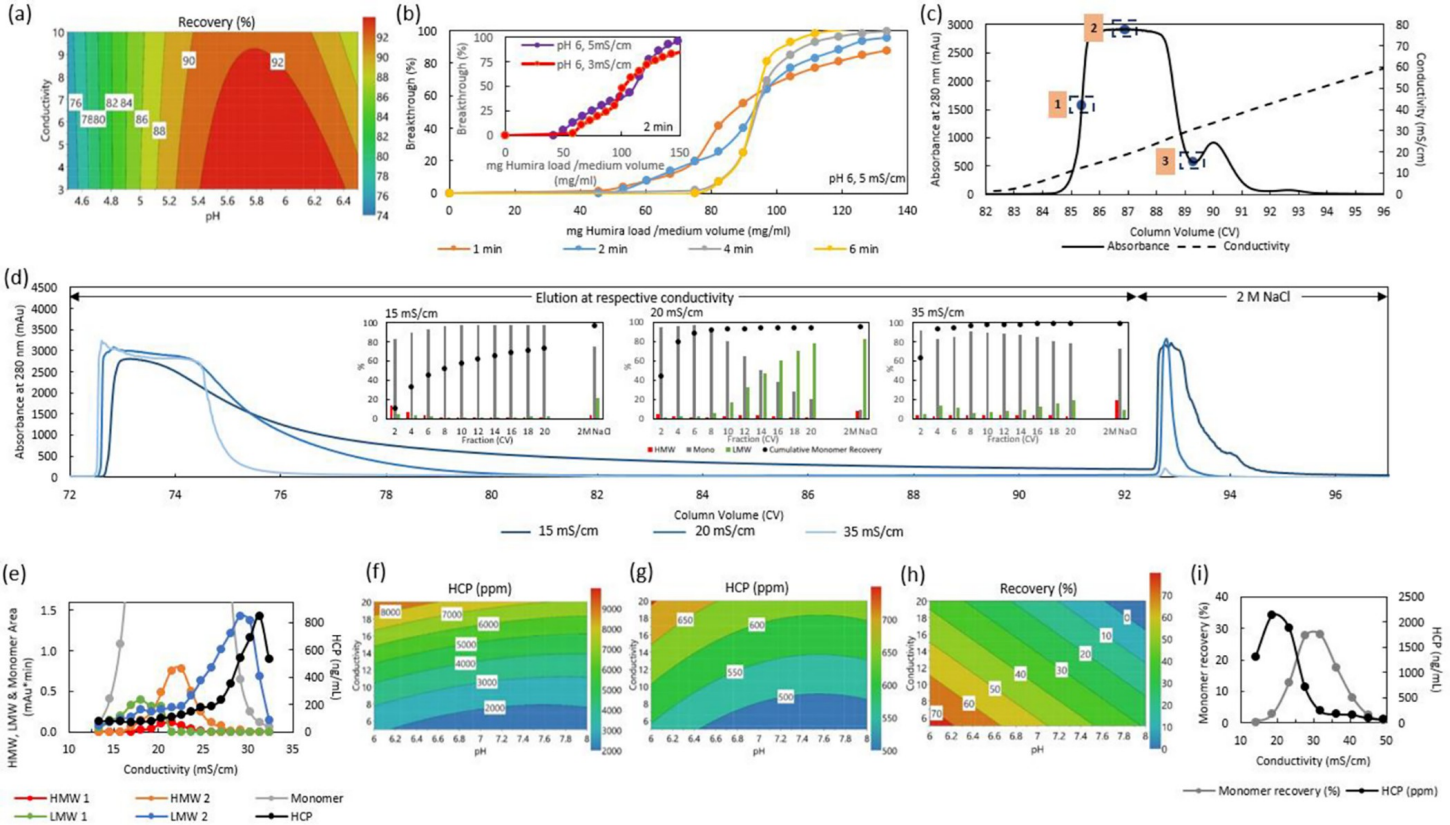
Optimisation of key process parameters for batch CEX capture and subsequent polishing steps. (a) 96-well plate screening of CEX load conditions. (b) Comparing the effect of different conductivities at pH 6, 2 min residence time (inset) and the effect of different residence times at pH 6, 5 mS/cm on the CEX loading breakthrough curves. (c) CEX AKTA gradient elution profile using 0–1 M NaCl 20 CV gradient elution at pH 6.0. (d) CEX AKTA step elution profile at 15 mS/cm, 20 mS/cm and 35 mS/cm, with the inset illustrating the relative percentage of HMW, LMW and monomeric species as well as the cumulative monomer recovery. (e) A detailed analysis of the profile of different subgroups of HMW, LMW, monomer species as well as HCP obtained using a 0–500 mM NaCl 20 CV gradient at pH 6.0. (f–h) 96-well plate screening of optimal conditions for polishing steps using AEX (f) and HIC resins (g) in FT mode, as well as optimal loading condition for multimodal chromatography resin (h). (i) The monomer and HCP elution profile obtained on the multimodal chromatography resin as a function of different conductivities obtained using 0 – 1 M NaCl 20 CV gradient elution at pH 6.0.

In order to develop the SMB CEX capture step protocol as well as to provide a basis of comparison, key process parameters were first optimized in batch mode. Using POROS XS (Thermo Fisher Scientific) as the model CEX resin, the optimal loading conditions were initially evaluated by condition screening in 96-well plate format at pH 4.5, 5.5, 6.5 and conductivity of 3, 6.5 and 10 mS/cm. The robust setpoint obtained from the contour plot was pH 6.0, conductivity 3 mS/cm (Fig 2A). As the loading time can be almost halved for a load of 5 mS/cm as compared to 3 mS/cm due to the required 4 and 7 X dilution factor respectively, the dynamic binding capacity (DBC) studies in 1 mL column format were evaluated at 2 min residence time for both conductivities (inset of Fig 2B). The DBC determined at 80% of 5% breakthrough were found to be similar at 49 g/L-resin (R) and 40 g/L-R for 3 mS/cm and 5 mS/cm respectively, hence the optimal loading pH and conductivity was determined as pH 6.0, 5 mS/cm. The effect of different residence times on the CEX loading capacity, as determined at 80% of 5% breakthrough, at the optimal loading condition was subsequently evaluated between the recommended residence times of 1–6 min [21]. Based on the breakthrough curves, a 4 min residence time resulted in a significant increase in loading capacity of 63 g/L-R compared to 44 g/L-R and 45 g/L-R for 1 min and 2 min residence times respectively (Fig 2B). As the loading capacity did not increase further and remained at 64 g/L-R at 6 min, the optimal residence time for loading was determined to be 4 min (Fig 2B).

The elution condition was next determined using a 0–1 M NaCl 20 column volumes (CV) gradient elution at pH 6.0. Based on the gradient elution profile, 3 conductivities– 15, 20 and 35 mS/cm–corresponding to the point of steepest UV increase, the mid-point of the main peak, and the valley separating the main peak and the shoulder peak (Fig 2C) were selected for subsequent 20 CV step elution validation (Fig 2D). Analysis of every consecutive 2 CV elution fraction reflects that the cumulative monomer recovery was the lowest (< 90%) for the 15 mS/cm elution, which was consistent with the massive tailing observed in the AKTA 280 nm absorbance signal (Fig 2D). Both 20 and 35 mS/cm elution gave similar high recoveries, with the 20 mS/cm elution condition yielding a clearer separation between the monomer and LMW species at earlier and later eluting fractions (Fig 2D). pH 6.0, 20 mS/cm was therefore selected as the optimal elution condition.

It is interesting to note that a shoulder peak that eluted at higher conductivities was clearly observed during the 0–1 M NaCl 20 CV gradient elution (Fig 2C), suggesting the presence of a subgroup of impurities that remained more strongly bound to the CEX resin compared to the target. This is also corroborated with the 20 mS/cm step elution profile which shows LMW species eluting at later fractions compared to the target monomeric species (Fig 2D). In order to further ascertain the elution profile of the different species, a 0–500 mM 20 CV NaCl gradient elution at pH 6.0 was performed, with the different subgroups of HMW and LMW species determined by HPLC-SEC based on retention times and HCP species determined by ELISA. In this way, it was observed that the LMW 2 elution profile closely matches that of the HCP, and that they remained more strongly bound to the CEX resin compared to that of the monomer (Fig 2E). The ability to enhance the separation between the strongly bound HCP species and the target using an advanced SMB configuration will be advantageous in enhancing target purity in a non-affinity capture step. As it was observed that the LMW 1 species and shoulder peak of the LMW 2 species eluted at ~17.5 mS/cm (Fig 2E), this was selected as the conductivity of the intermediate salt wash where weakly bound impurities can be removed. Based on the above determined batch parameters, the timing of each step of the SMB operation were optimized, as summarized in Table 1, to obtain the optimal target recovery and purity.

### 3.2 Development of polishing steps in batch mode for evaluation purposes

In order to determine the effect of the advanced SMB CEX approach on the overall process, three polishing resins with different modes of separation, namely POROS HQ (Thermo Fisher Scientific) as the model AEX resin, Benzyl Ultra (Thermo Fisher Scientific) as the model HIC resin, and Capto MMC ImpRes (Cytiva) as the model multimodal resin, were selected for protocol development. Although Capto MMC ImpRes is a multimodal cation exchanger, its surface functionality as a cation exchanger is a carboxylic group, while the surface functionality of POROS XS used as capture step is a sulfopropyl group. Moreover, as a multimodal cation exchanger, Capto MMC ImpRes works as an interplay of cation exchange, hydrophobic interaction, hydrogen bonding and thiophilic interaction. Therefore, it is possible that Capto MMC ImpRes could remove certain species that POROS XS could not. Condition screening studies in 96-well plate format were designed, using conditions at pH 6.0, 7.0, 8.0 and conductivity of 5, 12.5 and 20 mS/cm, with a load of 25 mg/mL-R. The batch CEX eluate was used for process parameters optimization here, so as to not create biasness, if any, towards the SMB CEX eluate.

For AEX and HIC resins employed in the FT mode, the lowest HCP was obtained at low conductivity and intermediate to high pH, as can be seen in the contour plots obtained from DoE software (Fig 2F and 2G). The robust setpoint determined in this way was at a conductivity of 6.0 mS/cm for both resins and pH 7.5 and pH 6.7 for the AEX and HIC resin respectively. During column validation runs, it was noted that while a 2 CV wash step was sufficient for the UV signal of the AEX to return to close to baseline, a 10 CV wash was necessary for the HIC resin, likely due to partial interactions between the target and the resin. For the multimodal resin, the robust setpoint of the loading condition was determined as pH 6.0, with a conductivity of 5.0 mS/cm (Fig 2H). The optimal elution condition was then determined by performing a 0–1 M NaCl 20 CV gradient elution at pH 6.0 in a 1 mL column (Fig 2I). By monitoring the monomer and HCP elution profile as a function of conductivity, it was observed that the HCP eluted at lower conductivities compared to the monomer. A 5 CV wash step at 20 mS/cm was therefore introduced, along with a 10 CV elution step at 34 mS/cm during column runs. A summary of the batch protocols are provided in Table 2, with a streamlined protocol proposed where pH 7.0, 6 mS/cm is employed for AEX and HIC FT polishing steps and multimodal chromatography load step so that no sample adjustment will be required.

**Table 2:** Process parameters of CEX, AEX, HIC and multimodal chromatography batch protocols. A residence time of 2 min was used for all steps unless otherwise stated.

Batch protocols		Equilibration	Load	Wash	Elution	Strip	Sanitization
**CEX** **(POROS XS)**	**CEX** **(POROS XS)**	50 mM MES, pH 6.0, 5 mS/cm, 3 CV	50 mM MES, pH 6.0, 5 mS/cm; 4 min residence time; Capacity: 63 g/L-R,	50 mM MES, pH 6.0, 17.5 mS/cm, 2 CV	50 mM MES, pH 6.0, 20mS/cm, 8 CV	2 M NaCl, 5 CV	1 M NaOH, 5 CV
**AEX** **(POROS HQ)**	**Optimal**	50 mM HEPES, pH 7.5, 6 mS/cm, 3 CV	50 mM HEPES, pH 7.5, 6 mS/cm, Evaluated up to: 51 g/L-R	50 mM HEPES, pH 7.5, 6 mS/cm, 2 CV		2 M NaCl, 5 CV	1 M NaOH, 5 CV
	**Streamlined**	Same as above, with the exception of pH 7.0					
**HIC** **(Benzyl Ultra)**	**Optimal**	50 mM MES, pH 6.7, 6 mS/cm, 3 CV	50 mM MES, pH 6.7, 6 mS/cm, Evaluated up to: 10 g/L-R	50 mM MES, pH 6.7, 6 mS/cm, 10 CV		1 M NaOH, 5 CV	
	**Streamlined**	Same as above, with the exception of pH 7.0					
**Multimodal** **(Capto MMC ImpRes)**	**Optimal**	50 mM MES, pH 6.0, 5 mS/cm, 3 CV	50 mM MES, pH 6.0, 5 mS/cm; Evaluated up to: 7.5 g/L-R	50 mM MES, pH 6.0, 20 mS/cm, 5 CV	50 mM MES, pH 6.0, 34 mS/cm, 10 CV	2 M NaCl, 5 CV	1 M NaOH, 5 CV
	**Streamlined**	Same as above, with the exception of pH 7.0, 6mS/cm					

Based on these developed polishing protocols, a suitable 2-step polishing strategy that can be effectively employed for the SMB CEX eluate was evaluated. When a complete FT mode strategy of AEX and HIC polishing steps was evaluated, it was observed that the HCP remained > 100 ppm even though the HMW species was effectively reduced to < 1% in both streamlined and optimal protocols (Strategy 1 and 2, Table 3). When AEX and multimodal chromatography were used as polishing steps with all FT and load conditions fixed at the streamlined conditions of pH 7.0 and 6 mS/cm, the HCP level was reduced to < 100 ppm but the HMW species remained > 1% (Strategy 3, Table 3). By utilizing HIC and multimodal chromatography as polishing steps at the streamlined conditions of pH 7.0 and 6 mS/cm, the HMW species and HCP level were successfully reduced to < 1% and < 100 ppm respectively within 2 polishing steps (Table 4).

**Table 3:** Evaluation of suitable polishing steps for SMB CEX eluate. All polishing runs were performed with 1 mL columns.

		Monomer recovery (%)	Monomer Purity (%)	HMW 1 (%)	HMW 2 (%)	LMW 1 (%)	LMW 2 (%)	HCP (ppm)
**SMB CEX Eluate** **(4 mL resin)**		94.1	96.9	0.1	1.4	0.0	1.6	4769
**Strategy 1** **(streamlined)**	**Post AEX FT**	99.7	97.3	0.1	1.4	0.0	1.2	1301
	**Post HIC FT**	97.8	99.1	0.0	0.2	0.0	0.7	291
**Strategy 2** **(optimal)**	**Post AEX FT**	99.5	97.4	0.1	1.4	0.0	1.2	1248
	**Post HIC FT**	97.4	99.0	0.0	0.4	0.0	0.7	277
**Strategy 3** **(streamlined)**	**Post AEX FT**	99.7	97.3	0.1	1.4	0.0	1.2	1301
	**Post Multimodal Eluate**	94.0	98.3	0.1	1.3	0.0	0.4	62

**Table 4:** Head to head comparison of the full purification process utilizing the SMB and batch CEX operations. All CEX runs were performed with a total of 4 mL resin and all polishing steps with 1 mL resin.

			Step Monomer recovery (%)	Overall monomer recovery (%)	Monomer Purity (%)	HMW 1 (%)	HMW 2 (%)	LMW 1 (%)	LMW 2 (%)	HCP (ppm)	hcDNA (ppm)
**CCS**			-	-	72.4	9.7	2.8	0.0	15.1	100222	420
**Post-PDK5**			90.5	90.5	82.0	1.7	1.8	0.0	14.5	96507	1
**SMB CEX**	**SMB CEX Eluate**		94.1	85.2	96.9	0.1	1.4	0.0	1.6	4769	< 0.1
	**Post HIC FT**		97.1	82.7	98.9	0.0	0.3	0.0	0.8	1122	< 0.1
	**Post Multimodal Eluate**		93.1	77.0	99.5	0.0	0.3	0.0	0.2	49	< 0.1
**Batch CEX**	**Batch CEX Eluate**		90.0	81.5	96.9	0.2	1.4	0.0	1.5	5154	<0.1
	**Post HIC FT**		97.0	79.0	98.2	0.0	0.6	0.0	1.3	1319	<0.1
	**Post Multimodal Eluate**		93.3	73.7	99.1	0.0	0.6	0.0	0.2	150	<0.1
	**Post AEX FT**		97.0	71.5	99.5	0.0	0.5	0.0	0.0	15	<0.1

### 3.3 Comparison of the full purification process utilizing SMB and batch CEX eluate

A final head-to-head comparison of the full purification process was performed, where all conditions, including total resin volume, load amount, flow rates and wash conditions, utilized in both SMB and batch CEX operations were aligned for a fair comparison (Table 4). It was observed that the SMB CEX eluate had a higher purity in terms of slightly lower HCP levels, as compared to that of the batch CEX eluate. Upon the application of the same 2-step polishing strategy on the batch CEX eluate as that applied on SMB CEX eluate, HCP levels remained > 100 ppm and required an additional AEX step in order to obtain HCP levels < 100 ppm. The fact that three polishing steps were necessary for the batch CEX eluate to meet therapeutic requirements whereas two polishing steps were sufficient for the SMB CEX eluate demonstrates the superior ability of the SMB CEX system at removing HCP, which represents a group of stronger binding impurities in the CCS used in this study. As the seemingly small difference in HCP levels between the SMB and batch CEX eluate resulted in the elimination of 1 polishing step, this may suggest that in addition to the absolute amount, the type of HCP species removed may also be critical. It is also important to note that the improvement in target purity did not occur at the expense of recovery. In fact, the step recovery of the SMB CEX eluate was ~ 4% higher than that of the batch CEX eluate. The higher step recovery of the SMB CEX eluate as well as the reduction of 1 polishing step led to ~ 6% higher overall process recovery using the SMB CEX eluate, compared to the corresponding process that utilized the batch CEX eluate. This highlights the ability of the advanced SMB approach to improve both target purity as well as recovery.

### 3.4 Future application for bispecific antibodies

Asymmetric IgG like bispecific antibody (bsAb) is the most popular bsAb design format which accounts for more than 50% of all bispecific antibodies that are currently under clinic trials. A knob-into-hole approach is the most common strategy employed to favour the formation of the target heterodimer bsAb over the mispaired products in the generation of asymmetric IgG-like bsAbs. However, homodimerization can still occur at low levels, with the occurrence of a certain amount of hole-hole homodimer and lower-level knob-knob homodimer impurities. Typically, the hole-hole homodimer and knob-knob homodimer impurities are both structurally and biophysically similar to their target bsAb monomers. In addition, a high level of aggregates and various antibody fragments are also common product-related impurities of bsAbs. All of these impurities are normally challenging to be removed or are removed with compromised target bsAb monomer recovery via batch mode chromatography and classical SMB system, and this could potentially be addressed by the advanced SMB approach. Taking Protein A chromatography as a capture step for example, based on their differences in binding avidity towards the Protein A ligand, aggregates are considered as strongly bound impurities, while certain hole-hole homodimers and antibody fragments could be considered as weakly bound impurities compared to bsAb monomers [22]. Leveraging the same advanced SMB system described in this paper, both strongly bound impurities (aggregates) and weakly bound impurities (hole-hole homodimersand antibody fragments) could potentially be separated from bsAb monomers via one Protein A capture step in a manner which could not be achieved by classical SMB. Moreover, although a portion of monomer bsAb could be co-eluted with weakly bound species during wash step, the leakage of monomeric bsAb will be continuously captured by the next Protein A column, therefore, the bsAb monomer recovery will be significantly improved compared to batch mode Protein A chromatography [22].

## 4. Conclusion

In conclusion, we present here the use of an advanced SMB approach that is capable of achieving a 3-component separation for the establishment of an effective non-affinity CEX capture platform technology. As a proof-of-concept, we demonstrate the enhanced removal of strongly bound host cell proteins (HCP) from the target mAb in a CEX capture step, leading to a reduction of 1 polishing step in order to meet therapeutic requirements of < 100 ppm HCP and an improvement of ~ 6% in overall process recovery compared to the corresponding process utilizing a batch operation. Another advantage of the advanced SMB approach presented here is that it offers the possibility for a further increase in load amount, which will lead to a greater utilization of the resin. While a mAb CCS has been utilised here, this approach is expected to be applicable to other challenging biotherapeutic modalities, such as for the removal of large amounts of aggregates associated with bispecific antibodies.
